# Validation of web-based remote photoplethysmography for heart rate measurement using standardized online infrastructure against ECG benchmarks

**DOI:** 10.3758/s13428-026-03098-7

**Published:** 2026-07-27

**Authors:** Gianluca Finotti, Daniele Di Lernia, Manos Tsakiris

**Affiliations:** 1 School of Psychological Sciences, Birkbeck, University of London, Malet St, London, WC1E 7HX UK; 2 Department of Theoretical and Applied Sciences, Research center in applied psychology (CePsi), eCampus University, Novedrate, CO 22060, Italy; 3 Applied Technology for Neuro-Psychology Lab, IRCCS Istituto Auxologico Italiano, Milan, Via Magnasco, 2, 20149, Italy; 4 Lab of Action and Body, Department of Psychology, Royal Holloway, University of London, Egham Hill, Egham TW20 0EX UK; 5 Centre for the Politics of Feelings, School of Advanced Study, University of London, London, UK

**Keywords:** rPPG, Heart rate, Remote photoplethysmography, Web-based physiological monitoring, Gorilla experiment builder, Interoception

## Abstract

Remote photoplethysmography (rPPG) enables non-invasive heart rate (HR) measurement via video, offering a promising tool for remote physiological monitoring. In a previous study, we validated an adapted rPPG algorithm developed by van der Kooij and Naber (Behav Res Methods 51(5):2106–2119, 2019), collecting video via an online platform using a common web browser and comparing results to phone-based HR measurements. In the present study, we extend this methodological pipeline by validating the same refined algorithm and online video collection pipeline against electrocardiogram (ECG) ground-truth measurements. While previous studies have typically relied on online data collection without ECG reference standards or laboratory-based protocols lacking online implementation, our study bridges this gap by directly comparing video data collected through a standard web browser using the Gorilla Experiment Builder platform with simultaneous ECG recordings. We also evaluated the optimal conditions for rPPG accuracy in terms of video duration and distance from the webcam. We recorded 180 videos across five durations (10 s, 25 s, 35 s, 45 s, and 100 s) and two distances (close = 35 cm, far = 50 cm). Results showed excellent agreement between rPPG- and ECG-derived HR, with an intraclass correlation coefficient (ICC) of 0.96 and 95% of measurements within Bland–Altman limits of agreement. Longer video durations significantly reduced error and variability, while recording distance had no significant effect. Validation of this rPPG pipeline against ECG ground truth establishes the reliability of web-based rPPG measurements, providing a foundation for large-scale online data collection with fidelity comparable to controlled laboratory assessments.

**Supplementary Information:**

The online version contains supplementary material available at https://doi.org/10.3758/s13428-026-03098-7.

## Introduction

Remote photoplethysmography (rPPG) enables non-invasive heart rate (HR) measurement through video recordings, eliminating the need for direct skin contact. Photoplethysmography relies on a simple principle: each heartbeat pumps blood to the periphery of the body, causing fluctuations in tissue haemoglobin levels and deforming the capillary bed in the skin (Mendelson & Kent, 1989; Teplov et al., 2014). These subtle changes in skin colouration, imperceptible to the naked eye, are captured in video frames and analysed to extract HR information.

Traditional plethysmography relies on contact sensors, such as finger or ear probes, to measure blood volume changes in localized areas of the body (Dorlas et al., 1985; Takano & Ohta, 2007). These sensors measure variations in light refraction inside the tissue, contingent on haemoglobin levels and capillary deformation, to measure the cardiac cycle.

In contrast, rPPG uses optical imaging to remotely detect these changes, offering significant advantages in terms of convenience and applicability. Specifically, it analyses video recordings of a person’s face with computer-based algorithms which detect changes in the amount of light reflected on the skin surface (e.g., Wang et al., 2021).

Numerous studies have demonstrated the effectiveness of this technique for measuring HR. In particular, Verkruysse and colleagues (2008) demonstrated that heart rate measurements could be derived through remote detection of subtle light variations on the skin using digital cameras.

Later advancements have led to open-source implementations of rPPG technology (Boccignone et al., 2020; McDuff & Blackford, 2019; van der Kooij & Naber, 2019). However, the accuracy of rPPG is significantly influenced by various factors, including ambient light conditions, the quality of the camera, the frame rate of video recordings, and the extent of body movement. To address these challenges, in a recent work (Di Lernia et al., 2024) we tested an algorithm developed by van der Kooij and Naber (2019), designed to perform reliably under suboptimal conditions, specifically on videos recorded via an online platform. We tested this algorithm using a validated dataset (CohFace) commonly employed for rPPG testing (Heusch et al., 2017), and on 231 videos recorded via the Gorilla platform (https://gorilla.sc/). The results demonstrated that this rPPG extraction procedure exhibits good reliability even when applied to videos collected via an online video recording platform. A key limitation of that study, however, was that rPPG-estimated HR was compared against HR measurements obtained using a mobile heart rate application. Although this application has been validated and shown to have good reliability and concurrent validity (Losa-Iglesias et al., 2016), it cannot be regarded as an absolute benchmark due to its inherent, albeit small, margin of error in HR estimation.

In the present study, we aimed to validate the accuracy of our rPPG extraction procedure using a methodological pipeline in which video data were systematically collected through an online recording platform while concurrently acquiring electrocardiogram (ECG) measurements as the physiological ground-truth reference standard. This methodological approach, which integrates online platform-based video acquisition with laboratory-controlled ECG validation, represents a significant advancement in establishing the psychophysiological fidelity of online-collected physiological data, thereby establishing the viability of platform-facilitated remote physiological monitoring with demonstrated concordance to clinical-grade cardiac measurements. Additionally, we examined the algorithm's performance under various conditions to determine how video duration and the distance between the webcam and the participant affect accuracy. Identifying these parameters is essential for maximizing the likelihood of obtaining accurate HR estimates in uncontrolled, real-world experimental settings. To further preserve ecological validity, lighting conditions were not standardized using artificial or fixed light sources. Instead, all recordings were conducted using naturally available daylight, across different hours of the day, reflecting the kind of environmental lighting participants might encounter when completing experiments outside a laboratory (e.g., at home). This design choice allowed us to evaluate the algorithm’s robustness under ecological, non-controlled conditions while still ensuring a minimum threshold of visibility for accurate signal extraction.

## Methods

### Participants

Eighteen participants took part in the study (12 female, 6 male, *M*_age_ = 26.27 years, *SD* = 13.24, range = 18–72). All participants provided written informed consent before participation. The study was approved by the Ethics Committee at Royal Holloway University of London.

### Procedure

The experimenter placed three disposable pre-gelled ECG electrodes (Ag/AgCl) on participants in a modified lead III chest configuration. Two electrodes were positioned below the left and right collarbones, while the third electrode was placed on the lower left side of the back. The ECG signal was recorded using the PowerLab 8/35 (ADInstruments, http://www.adinstruments.com/) with LabChart 8 Pro software. The sampling rate was set to 1,000 Hz, and a hardware band-pass filter (Bio Amp 132) between 0.3 and 1,000 Hz was applied. Heartbeats were detected online using a hardware-based function that identifies the ECG R-waves when the signal amplitude exceeds a participant-specific threshold.

Participants were seated at a table facing a computer monitor equipped with a Logitech C920 HD webcam mounted on its upper edge. This full HD webcam is capable of recording at 1080p resolution with a frame rate of 30 frames per second and features a 78° diagonal field of view.

We used Gorilla Experiment Builder to create and host our experiment (Anwyl-Irvine et al., 2020). We used the beta video recording zone functionality to activate the webcam and record videos of the participant’s face. This setup ensured that the rPPG could be evaluated on videos comparable in quality to those recorded during remote experiments on the same platform. Participants were informed when the video recording started and ended with an audio cue (i.e., a 200 ms beep). During recordings, a black fixation cross was displayed on a white background, and participants were instructed to focus on the cross while minimizing head movement. However, natural blinking was permitted. After each recording, participants could take a short break and advance to the next trial by pressing a key on the keyboard.

To simulate real-world recording conditions and enhance ecological validity, lighting was not artificially controlled or standardized using fixed light sources. Instead, all video recordings were conducted using naturally available daylight at various times throughout the day. This approach reflects the type of ambient lighting participants are likely to encounter in home-based, remote experiments. While no artificial lighting setup was used, a minimum quality threshold was maintained: if ambient light was deemed insufficient by the experimenter (e.g., during very low light), the recording session was rescheduled to ensure the participant’s face was clearly visible with minimal shadows or overexposure. This balance allowed us to preserve naturalistic conditions while still ensuring the facial visibility required for reliable rPPG signal extraction.

Each participant’s session included 10 video recordings: five durations (10 s, 25 s, 35 s, 45 s, and 100 s) recorded at two distances (close = 35 cm, and far = 50 cm). The order of conditions was counterbalanced across participants to minimize order effects. These durations were chosen a priori to span a practically useful range of brief recording windows for online psychological research, from very short to relatively longer acquisitions, while remaining feasible within an experimental session. We focused on short windows because they are often easier to integrate into online task designs, whereas recordings lasting several minutes may be less practical in many paradigms because they require more time from participants, may increase fatigue or boredom, and can interrupt task flow.

To synchronize the videos with the ECG recordings, we used a photodiode (that is, a light-sensitive semiconductor) controlled with an Arduino Uno. At the start and the end of each trial, a 2 × 2 cm black square appeared in the bottom right corner of the screen. The photodiode, placed on the screen, detected changes in light intensity caused by the appearance of the square. Upon detection, the Arduino triggered an event marker on the ECG trace in LabChart, enabling precise alignment of the ECG data with the start and end of each video recording.

### Data cleaning

All videos were processed with our rPPG algorithm. As a first step, we checked that all videos had a frame rate (FR) greater than or equal to 20. As all the videos met this criterion, none were removed due to low frame rate. Next, we checked whether the real HR and the rPPG HR were between 50 and 120 beats per minute (BPM). All real and estimated HR fell within this range. Finally, we used the R *boxplot* function to detect and remove outliers, defined as the values outside the interquartile range, for both the real HR and the rPPG HR. The rPPG HR estimated on one video was detected as outlier and removed from further analysis. After these preprocessing steps, 179 videos from 18 participants were retained for the final analysis (one excluded).

### rPPG extraction

The rPPG extraction was conducted using an approach previously described in detail by Di Lernia and colleagues (2024). This method utilizes video recorded by an online platform (Gorilla Experiment Builder) and leverages MATLAB to process video frames and extract heart rate signals based on fluctuations in facial skin colour. Briefly, the method includes converting online-collected data to preserve ppg information, detecting facial features using a cascade object detector, tracking feature points with the Kanade–Lucas–Tomasi algorithm, and localizing skin regions for analysis. Average RGB values of skin pixels are extracted and resampled to 60 Hz. The data are then band-pass filtered, dimension-reduced using the plane-orthogonal-to-skin (POS) algorithm and analysed via a Lomb–Scargle periodogram to derive signal-to-noise ratios (SNR) for heart rate estimation.

For a comprehensive description of the rPPG extraction steps, including modifications to improve the algorithm's accuracy, refer to Di Lernia and colleagues (2024).

### ECG processing

The ECG traces were segmented and matched to the corresponding videos. Then, we visually inspected the traces to ensure that the R-peaks had been correctly detected by the fast-response output function, and we used a custom MATLAB script to count the number of R-peaks. Finally, we used the number of beats and the trial duration to calculate the real heart rate during each trial.

### Analysis

First, we assessed whether the variables of interest (ECG HR and rPPG HR) were normally distributed. This was done by visual inspection of quantile–quantile plots (Q–Q plots), which compare the quantiles of the sample data to a theoretical normal distribution, as well as by examining skewness (a measure of asymmetry in the data) and kurtosis (a measure of the “tailedness” of the distribution). Visual inspection indicated that the data were approximately normally distributed.

To evaluate the concurrent validity of the rPPG extraction pipeline compared to ECG, we calculated the intraclass correlation coefficient (ICC) to assess the agreement between the two methods. The ICC was computed using a two-way random-effects model (ICC[A,1]) with an agreement definition, which assumes that both the measurement methods (rPPG and ECG) and the subjects are random effects. This approach is widely used for assessing absolute agreement between methods and is consistent with prior studies (Losa-Iglesias et al., 2016). The ICC was calculated in R using the icc function from the irr package with the following parameters: icc(data, model = “twoway”, type = “agreement”, unit = “single”). This model considers single measurements (rather than averages) and evaluates the degree to which the two methods produce the same values across subjects.

Next, we quantified the agreement between the two methods by calculating the limits of agreement (LoA) using Bland–Altman statistics (Bland et al., 1999). This is one of the most common methods in clinical research and medical laboratories for determining whether two measurement methods are within a clinically acceptable difference (e.g., Giavarina, 2015; Karun & Puranik, 2021). The LoA can be used to build a Bland–Altman plot, which makes it easy to investigate whether there is a systematic difference between two measurements.

This plot shows the mean of the difference and the 95% LoA, marked by the lower limit of agreement (LL = mean of difference − 1.96 × standard deviation of difference) and the upper limit of agreement (UL = mean of difference + 1.96 × standard deviation of difference). The closer the datapoints are to the zero line, the greater the agreement between the two methods. Also, if the constructed limits of agreement are within a range that is considered acceptable, the two methods can be used interchangeably. Whether the limits are considered acceptable depends on the measure being tested and its intended application. For example, methods used in clinical practice, where small errors may have serious consequences, require narrower LoA. Bland–Altman statistics were calculated using the *blandr* package for R with a 0.95 significance level and “mode” = 1, which calculates the LoA with the more accurate 1.96 multiplier. To further test the relation between the ECG HR and the rPPG HR, we performed a Pearson correlation.

To explore systematic bias, we employed Passing–Bablok regression (Bilic-Zulle, 2011), a robust non-parametric method designed to evaluate potential systematic and proportional bias between two measurement techniques. Here, rPPG HR was treated as the test method and ECG HR as the reference method.

Passing–Bablok regression identifies two types of systematic bias: constant bias, reflected in the intercept, and proportional bias, reflected in the slope. A significant intercept would indicate a constant bias, where one method consistently overestimates or underestimates values compared to the other. A slope differing significantly from 1 would suggest proportional bias, indicating that discrepancies between the methods vary with the magnitude of the measurement. By applying this approach, we assessed whether rPPG HR measurements systematically differed from ECG HR measurements and whether any potential bias was dependent on the heart rate's magnitude. All previous analyses were conducted in R (version 4.3.2, 2023–10–31 ucrt). Detailed information on all attached and namespace-loaded packages, including their versions, is provided in the appendix to ensure reproducibility.

Finally, we calculated the absolute difference between ECG HR and rPPG HR scores and we ran a 2 × 5 repeated-measures analysis of variance (ANOVA) on these scores. This analysis asked whether there was a difference in the rPPG HR error (that is, the difference between the estimated HR and real HR) depending on the distance of the participant from the webcam (35 cm or 50 cm) or the duration of the videos (10 s, 25 s, 35 s, 45 s, 100 s). The two factors were distance (two levels: 35 cm, 50 cm) and duration (five levels: 10 s, 25 s, 35 s, 45 s, 100 s). The dependent variable was the absolute difference between ECG HR and rPPG HR. This analysis was conducted in JASP (Love et al., 2019). An annotated.jasp file, including data and input options, is available at (https://osf.io/3ksjd/).

## Results

**Fig. 1:**
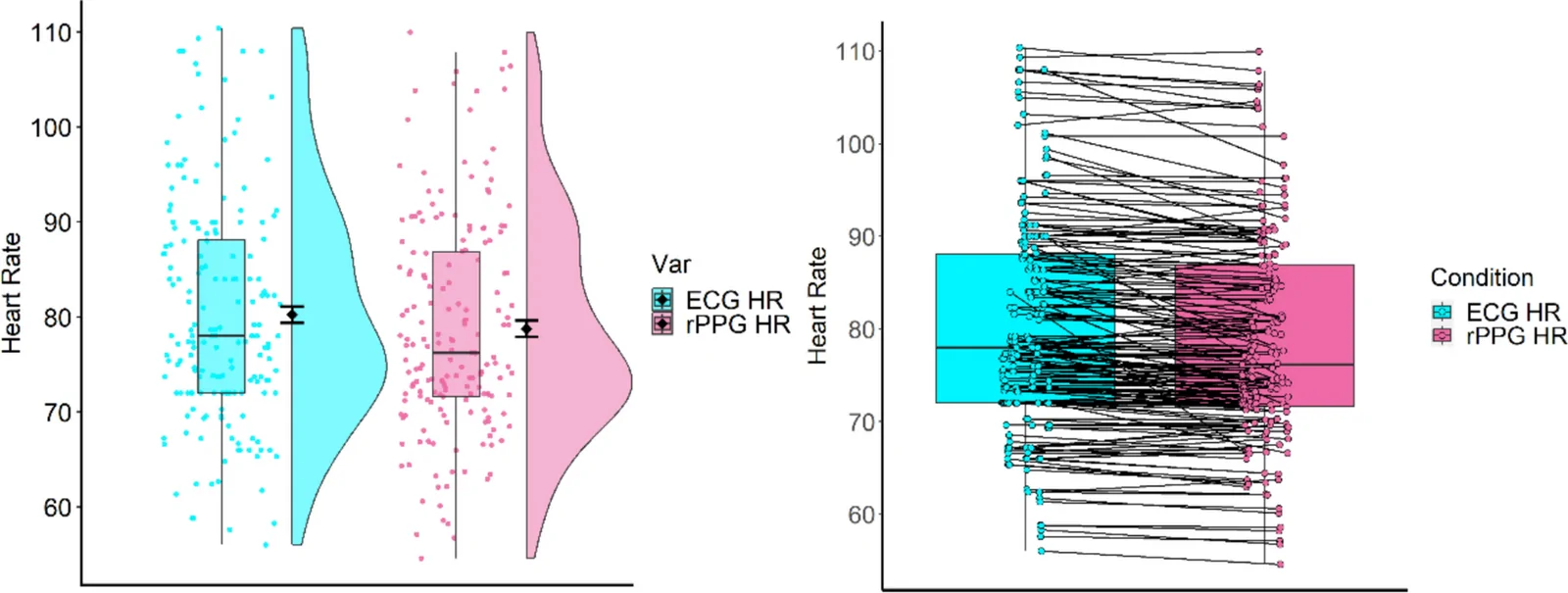
Left panel: Raincloud plots show data distribution, the central tendency by boxplots, and the jittered raw data for the two methods (ECG and rPPG HR). The vertical bars inside the distributions depict the mean and standard error. Right panel: Similar to the left panel, this shows the central tendency by boxplots and the jittered raw data, but here the HR extracted with the two different methods are interconnected for each video

Table 1 shows the descriptive statistics of the HR recorded with the ECG and rPPG (see also Fig. 1, left and right panels).

**Table 1:** Descriptive statistics for the HR recorded with the ECG and rPPG

	Mean	*SD*	IQR	Median	Range (min–max)
ECG	80.24	11.83	16.1	78	56–110.4
rPPG	78.74	11.45	15.25	76.18	54.56–109.96

*ECG*, electrocardiogram; *rPPG*, remote photoplethysmography; *SD*, standard deviation; *IQR*, interquartile range.

The results of the ICC analysis demonstrated excellent agreement between the rPPG and ECG measurements. The ICC value was 0.96 (95% CI [0.92, 0.98]), which falls within the “excellent” range of agreement (for the interpretation of the ICC, see Koo & Li, 2016). The *F*-test for the null hypothesis that the ICC equals zero was statistically significant, *F*(178, 23.5) = 59.5, *p* < 0.001, confirming the strong agreement between the two methods.

**Fig. 2:**
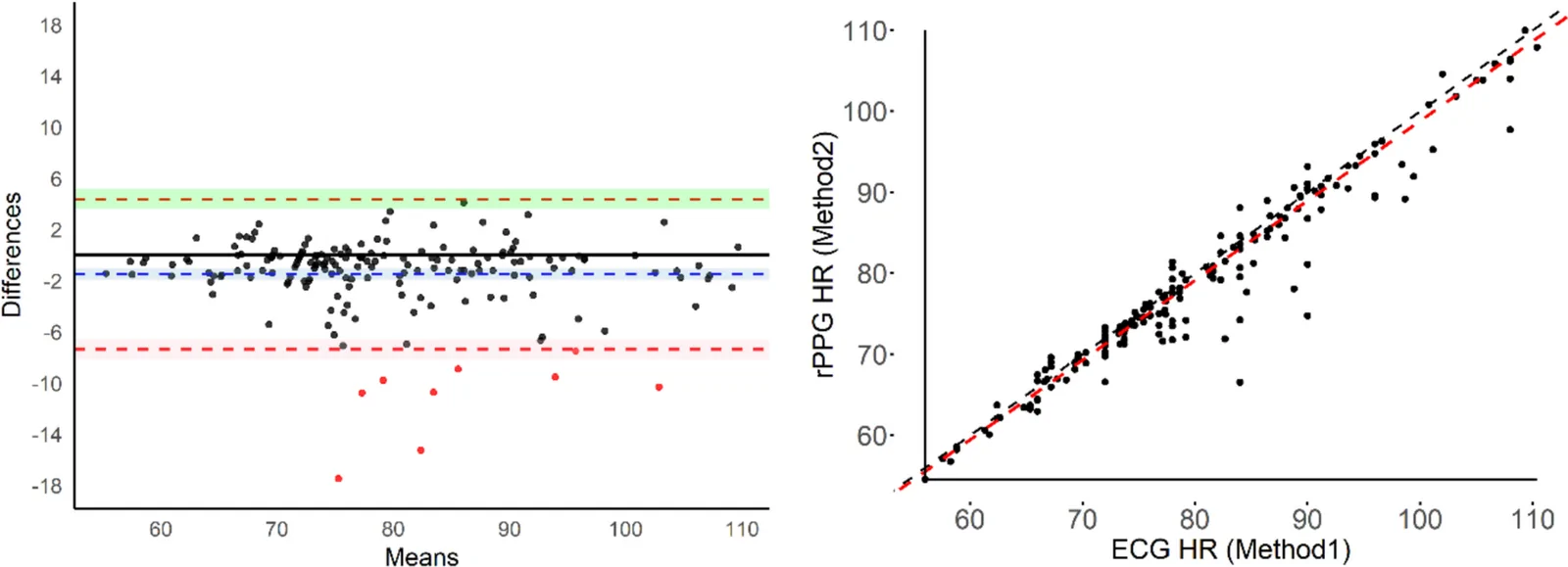
Left panel: Bland–Altman plot of differences between the ECG HR and rPPG HR scores (all timings and distances included), *y*-axis, versus the mean of the two measurements, *x*-axis. The blue dashed line represents the mean of the differences (bias), while the red dashed lines represent the 95% limits of agreement (LoA; upper limit = mean of difference + 1.96 SD, lower limit = mean of difference – 1.96 SD). The green and pink shaded areas show the confidence intervals (CI) for the upper and lower LoA, respectively, while the light blue shaded area represents the CI for the bias. Black dots represent data points that fall within the 95% LoA, whereas red dots highlight data points that lie outside these bounds. The bias is represented by the vertical gap between the *x*-axis (zero difference) and the blue dashed line. Right panel: Passing–Bablok regression plot comparing rPPG HR to ECG HR. The dashed red line represents the Passing–Bablok regression fit, while the dashed black line represents the identity line (slope = 1, intercept = 0), indicating perfect agreement. The regression fit demonstrates a near-perfect alignment with the identity line, with minimal systematic bias between the two methods

Bland–Altman statistics revealed a bias of − 1.49 BPM, indicating that the rPPG extraction pipeline tends to slightly underestimate heart rate compared to the ECG. The 95% confidence interval (CI) for the bias ranged from − 1.94 to − 1.05 BPM, with a standard error of bias (SEB) of 0.22 BPM. The limits of agreement (LoA) were − 7.36 BPM (95% CI [− 8.11, − 6.60]) and 4.37 BPM (95% CI [3.61, 5.12]). As shown in Fig. 2, left panel, 94.97% of the measurements fell within the 95% LoA, with only 5.03% of the points outside these bounds. This demonstrates that the rPPG extraction pipeline is a reliable and viable substitute for ECG in measuring heart rate.

Furthermore, a bootstrapped Pearson correlation revealed a strong, positive correlation between ECG HR and rPPG HR, which was statistically significant (*r* = 0.97, bias-corrected and accelerated [BCa] 95% CI [0.956, 0.975], *p* < 0.001).

The Passing–Bablok regression analysis yielded an intercept of 0.44 (95% CI [− 1.15, 2.24]) and a slope of 0.98 (95% CI [0.96, 1.00]). The intercept being close to zero indicates no substantial systematic error, while the slope close to 1 suggests negligible proportional bias. These findings suggest that rPPG HR provides a highly comparable estimation to ECG HR, with minimal bias.

The Pearson correlation coefficient was *r* = 0.967, further indicating strong agreement between the two methods (see Fig. 2, right panel). These findings suggest that rPPG HR provides a highly comparable estimation to ECG HR, with minimal bias.

The final analysis investigated whether the accuracy of the rPPG HR (measured as absolute difference with the real HR) was affected by the distance from the webcam and the duration of the video recording. The repeated-measures ANOVA showed that the main effect of distance was not significant, *F*(1, 16) = 0.154, *p* =.699, η_p_^2^ = 0.010, whereas the main effect of duration was significant *F*(2.16, 34.67) = 7.591, *p*_g-g_ =.001, η_p_^2^ = 0.32. Finally, the interaction between distance and duration was not significant, *F*(1.78, 28.58) = 2.11, *p*_g-g_ =.144, η_p_^2^ = 0.11.

**Fig. 3:**
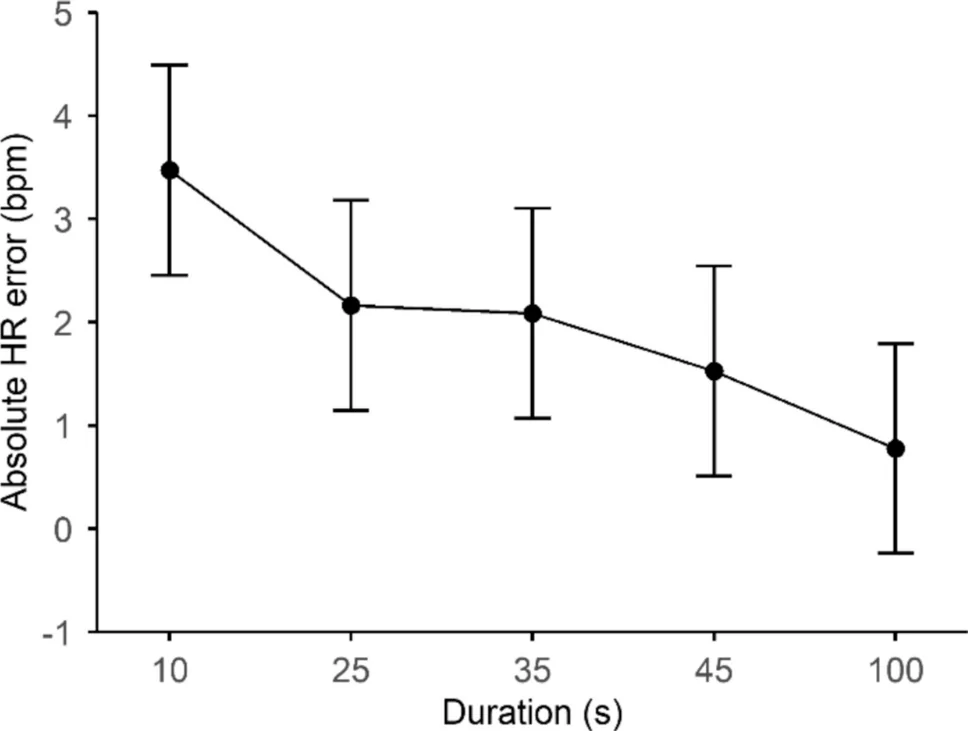
Estimated marginal means of rPPG HR error (absolute difference between rPPG HR and real HR) across video durations. Error bars represent 95% confidence intervals. The figure illustrates how rPPG HR error varies by video duration

Post hoc pairwise comparisons with a Bonferroni correction indicated that the 10-s videos had significantly higher errors than the 45-s (*p*_bonf_ = 0.003) and 100-s (*p*_bonf_ <.001) videos; all other comparisons were non-significant (see Tables 2 and 3 and Fig. 3).

**Table 2:** Post hoc tests comparing the rPPG HR estimation error between videos of different durations

			95% CI for mean difference				
Duration (s)		Mean difference	Lower	Upper	*SE*	*t*	*p*_bonf_
10	25	1.312	− 0.165	2.789	0.508	2.582	0.121
	35	1.387	− 0.090	2.865	0.508	2.730	0.082
	45	1.947	0.470	3.425	0.508	3.833	0.003
	100	2.694	1.217	4.172	0.508	5.303	<.001
25	35	0.075	− 1.402	1.553	0.508	0.148	1.000
	45	0.635	− 0.842	2.113	0.508	1.251	1.000
	100	1.382	− 0.095	2.860	0.508	2.721	0.084
35	45	0.560	− 0.917	2.038	0.508	1.102	1.000
	100	1.307	− 0.170	2.784	0.508	2.572	0.124
45	100	0.747	− 0.731	2.224	0.508	1.470	1.000

*SE*, standard error; *p*_bonf_, *p* values after Bonferroni correction.

**Table 3:** Descriptive statistics for the rPPG HR error (calculated as absolute difference between rPPG HR and real HR)

Duration	Mean error	SD error
10	3.40	4.10
25	2.15	2.13
35	2.14	2.18
45	1.48	2.26
100	0.81	1.29

*SD*, standard deviation.

## Discussion

The aim of this study was to validate the psychophysiological accuracy of our rPPG extraction pipeline when applied to video recordings acquired through standardized online platform infrastructure. We implemented a dual-modality assessment protocol in which videos recorded via the Gorilla platform in a common web browser were systematically compared against simultaneous electrocardiogram measurements. Our results demonstrate excellent agreement between rPPG- and ECG-derived heart rates, with an ICC of 0.96 and 95% of measurements falling within the Bland–Altman limits of agreement. This evidence represents a significant methodological advancement in remote physiological assessment by establishing a critical bridge between online data acquisition procedures and laboratory-grade psychophysiological measurement.

In a previous study (Di Lernia et al., 2024), we validated the rPPG algorithm against heart rate measurements obtained via a phone app. While that study demonstrated promising reliability, the use of a phone app as a reference introduces a margin of error inherent to the device itself. Conversely, the present study's unique contribution lies in specifically validating the measurement accuracy of videos acquired through online platform infrastructure against gold-standard ECG reference measurements. By validating against ECG in the current study, we have substantially strengthened the evidence for the online acquisition pipeline accuracy, providing a benchmark for future studies seeking to implement this rPPG algorithm in remote or uncontrolled environments.

A secondary aim of this study was to identify optimal conditions for achieving high rPPG accuracy.

We found that video duration significantly influenced rPPG error, with longer recordings (45 s and 100 s) yielding lower error rates than shorter recordings (10 s). This finding suggests that extended recordings allow for more reliable signal extraction and reduce variability due to transient noise. Interestingly, while the 25 s and 35 s durations did not significantly outperform the 10 s videos in post hoc tests, the descriptive trends suggest an incremental improvement in accuracy with increased duration. It should be noted that the present study was designed to evaluate performance across a practically relevant range of brief recording windows, rather than to determine the maximal useful recording duration, and we therefore cannot conclude whether recordings longer than 100 s might yield further gains in accuracy.

In contrast, the distance between the participant and the webcam did not significantly affect rPPG performance. This suggests that the algorithm's ability to extract heart rate signals is robust to variations in recording distance within the tested range (35 cm vs 50 cm). This finding is particularly encouraging for remote applications, where users may not consistently adhere to precise distance guidelines.

Taken together, these results show that our rPPG algorithm can be used to estimate the HR of participants taking part in online experiments via the Gorilla platform.

Compared with a previous validation study (Di Lernia et al., 2024), the present experiment shows higher accuracy of the rPPG. This improvement is most likely not due to technical differences in the hardware used but rather to better control over ambient conditions. In our previous work, participants were provided with detailed instructions, such as placing the recording device on a stable surface like a table, performing the experiment in a well-illuminated room with natural light, ensuring the face was properly lit but not overexposed, avoiding shadows or obstructions on the face, positioning the camera directly in front of the eyes, and minimizing body movement during the video recordings. Despite these guidelines, many participants recorded videos in suboptimal conditions, such as overexposed or poorly lit rooms, which likely contributed to increased variability in signal quality.

In the current study, the experimenter oversaw all recordings to ensure minimum quality standards, particularly avoiding very dark environments. Importantly, however, lighting was not controlled (no artificial light sources or fixed setups were used). Instead, videos were captured using only naturally available daylight, under typical ambient conditions. In fact, many recordings took place on overcast or moderately lit days, providing just enough illumination for the face to be clearly visible without harsh shadows or overexposure.

This approach enhances ecological validity, as it demonstrates that our extraction pipeline can provide high-quality rPPG signals under everyday non-studio conditions—conditions that are realistically achievable by participants at home when given clear and specific recording instructions. Emphasizing the importance of following these guidelines, such as ensuring proper lighting and avoiding obstructions or shadows, is critical to maintaining data quality. Additionally, videos that fail to meet these prerequisites should be excluded from analyses to preserve the reliability of the results.

The findings of this study highlight the practical utility of rPPG for remote heart rate monitoring, particularly in research settings where controlled experimental setups may not be feasible. The strong agreement between rPPG and ECG underscores the reliability of this extraction pipeline for non-clinical applications, such as psychological experiments. Furthermore, the minimal influence of recording distance on accuracy demonstrates the extraction pipeline’s flexibility in accommodating participants’ natural behaviours and varied setups.

At the same time, although agreement between rPPG and ECG was strong overall, some individual recordings showed larger discrepancies between the two methods. This is an important practical limitation, particularly for applications in which reliable estimation at the level of individual recordings is critical. Future studies should therefore investigate whether it is possible to identify recordings at increased risk of inaccuracy, for example through the development and validation of suitable internal quality-control indicators. Addressing this issue will be an important next step in the further development of the method.

For clinical applications where precision is critical, further testing and refinement will also be necessary. With the growing demand for remote tools to measure physiological signals (e.g., Tohma et al., 2021), the present work indicates that rPPG holds promising potential. Nevertheless, additional development and rigorous validation are required before this technique can be reliably used for clinical purposes.

An important limitation concerns participant-level generalizability. It is important to note that previous research has shown significant reductions in rPPG performance with darker skin tones (Nowara et al., 2020). This study did not include participants with darker skin tones, limiting the generalizability of the findings for broader online applications. Additionally, these studies have not yet systematically tested whether accuracy varies as a function of other participant characteristics such as age, sex, facial hair, race or ethnicity, or body mass index (BMI).

For these reasons, the current findings should not be interpreted as demonstrating equivalent performance across these characteristics.

Future studies should specifically examine how skin tone influences rPPG accuracy to ensure inclusivity and robustness in diverse populations. Additionally, the relatively small sample size of 18 participants, while adequate for demonstrating proof of concept, constrains the generalizability of the results. Future research should include larger and more demographically diverse samples to evaluate the algorithm’s performance across varied populations, particularly with respect to skin tone, physiological differences, and other relevant factors.

Second, although we controlled for some environmental factors, such as ensuring consistent natural lighting, we did not systematically test the algorithm under extreme or variable conditions (e.g., dim lighting, different webcams, or participant motion). Future research should explore the performance of the rPPG extraction pipeline in such scenarios to better replicate real-world applications. Finally, while we focused on video duration and distance, additional parameters such as webcam resolution and frame rate should also be systematically examined in future studies to identify the most important factors affecting rPPG accuracy.

In conclusion, this study validates the accuracy and reliability of our refined rPPG algorithm against ground-truth ECG measurements, demonstrating its potential for remote heart rate monitoring. Longer video durations significantly reduce rPPG error, while recording distance has minimal impact within the tested range. These findings highlight the importance of optimizing recording conditions to maximize the algorithm's utility in both research and practical applications. Future studies should focus on expanding the algorithm's robustness and testing its applicability under more diverse and uncontrolled conditions.

## Supplementary Information

Below is the link to the electronic supplementary material.

Supplementary file1 (PDF 46 kb)

## Data Availability

Data and analysis scripts supporting the findings of this study are openly available on the Open Science Framework (OSF) at: https://osf.io/3ksjd/. The original video recordings are not shared, as participants did not provide consent for their distribution.

## Code availability

The R script used for data analysis and an annotated JASP file (including data and input options) are available at the same OSF link.
