# Supplementary material for: Validation of web-based remote photoplethysmography for heart rate measurement using standardized online infrastructure against ECG benchmarks
Source: Behav Res Methods. 2026 Jul 27;58(9):252. doi: 10.3758/s13428-026-03098-7 (PMC13407969; doi:10.3758/s13428-026-03098-7)
Supplement: Supplementary file 1 — Supplementary file1 (PDF 46 kb) [file 13428_2026_3098_MOESM1_ESM.pdf]

Validation of web-based remote photoplethysmography for heart rate measurement using standardized online infrastructure against ECG benchmarks.

Appendix

Software and Package Information

To ensure transparency and reproducibility, we provide detailed information about the computational environment used for data analysis. This includes:

- A list of all **attached packages** and their respective versions
- A list of **additional packages loaded via namespace**, including their versions

This information is based on the output of the sessionInfo() function in R and reflects the exact environment in which the analyses were conducted.

attached base packages:

```
[1] parallel  stats     graphics  grDevices utils     datasets  methods   base
```

other attached packages:

```
 [1] MethodCompare_1.0.0 mcr_1.3.3.1         robslopes_1.1.3     blandr_0.6.0
 [5] irr_0.84.1          lpSolve_5.6.21      patchwork_1.3.0     ggpubr_0.6.0
 [9] Rmisc_1.5.1         lattice_0.21-9      plyr_1.8.9          reshape_0.8.9
[13] cowplot_1.1.3       MASS_7.3-60         writexl_1.5.0       tidyr_1.3.1
[17] tibble_3.2.1        psych_2.4.12        dplyr_1.1.4         ggplot2_3.5.1
[21] readxl_1.4.3
```

loaded via a namespace (and not attached):

```
 [1] gtable_0.3.5        xfun_0.45           rstatix_0.7.2       vctrs_0.6.5         tools_4.3.2
 [6] generics_0.1.3      fansi_1.0.6         pkgconfig_2.0.3     Matrix_1.6-1.1      RColorBrewer_1.1-3
[11] RcppParallel_5.1.9 jmvcore_2.6.3       lifecycle_1.0.4     compiler_4.3.2      farver_2.1.2
[16] stringr_1.5.1       munsell_0.5.1       mnormt_2.1.1        carData_3.0-5       htmltools_0.5.8.1
[21] yaml_2.3.8          pillar_1.9.0        car_3.1-2           crayon_1.5.3        lamW_2.2.4
[26] abind_1.4-5         nlme_3.1-163        tidyselect_1.2.1    digest_0.6.35       stringi_1.8.4
[31] reshape2_1.4.4      purrr_1.0.2         geepack_1.3.12      labeling_0.4.3      splines_4.3.2
[36] fastmap_1.2.0       grid_4.3.2          colorspace_2.1-0    cli_3.6.2           magrittr_2.0.3
[41] utf8_1.2.4          broom_1.0.6         withr_3.0.0         scales_1.3.0        backports_1.5.0
[46] rmarkdown_2.27      ggsignif_0.6.4      cellranger_1.1.0    evaluate_0.24.0     knitr_1.47
[51] mgcv_1.9-0          rlang_1.1.3         Rcpp_1.0.12         glue_1.7.0          jsonlite_1.8.8
[56] rstudioapi_0.16.0  R6_2.5.1
```

For Review Only
